# Preparation and Performance Investigation of Broadband Antireflection Coatings in the Visible and Near-Infrared Spectral Regions

**DOI:** 10.3390/mi17091077

**Published:** 2026-09-11

**Authors:** Zhaoxuan Zheng, Zaijin Li, Qi Wu, Fei Lin, Yishui Lin, Ganghuang Liu, Runhe Bai, Wei Luo, Dongxin Xu, Yi Qu

**Affiliations:** 1 Hainan Key Laboratory of Laser Technology and Optoelectronic Functional Materials, Collaborative Innovation Center for Flexible Talents Introduction, Hainan Normal University, Haikou 571158, China; 202213085400018@hainnu.edu.cn (Z.Z.); lizaijin@hainnu.edu.cn (Z.L.); 2025200057@mails.cust.edu.cn (Q.W.); 202413085408008@hainnu.edu.cn (F.L.); 202313085408006@hainnu.edu.cn (Y.L.); 202413085408009@hainnu.edu.cn (G.L.); 202412070200001@hainnu.edu.cn (R.B.); 202313085408009@hainnu.edu.cn (W.L.); jilinchangchun@yeah.net (D.X.); 2 Hainan International Joint Research Center for Semiconductor Lasers, School of Physics and Electronic Engineering, Hainan Normal University, Haikou 571158, China; 3 Hainan Hairuina Optoelectronics Co., Ltd., Haikou 571127, China; 4 National Key Laboratory of Semiconductor Laser, Changchun University of Science and Technology, Changchun 130022, China

**Keywords:** broadband antireflection coating, ion-assisted electron-beam evaporation, visible–near-infrared spectral range, thickness-tolerance analysis

## Abstract

A seven-layer broadband antireflection coating consisting of Al_2_O_3_, TiO_2_, SiO_2_, and MgF_2_ was designed and deposited on both sides of K9 glass for the 400–1100 nm range. The deposition parameters for TiO_2_ and SiO_2_ single layers were optimized, and the measured optical constants were used in TFCalc to refine the multilayer design. Layer-specific thickness-tolerance analysis was performed to identify the layers requiring strict process control. The initially characterized double-sided coating showed an average transmittance of 98.72% over 400–1100 nm. Four samples prepared in one additional deposition batch exhibited closely matching transmittance spectra, indicating low sample-to-sample variation within that batch. Five-position profilometry indicated consistent total-thickness control. Cross-sectional scanning electron microscopy (SEM) revealed well-defined multilayer contrast without obvious cracking, and regional energy-dispersive X-ray spectroscopy (EDS) analysis detected the constituent elements of the coating. These results demonstrate a practical design-to-fabrication approach that combines process-specific optical constants with tolerance analysis, with potential applications in imaging systems, optical windows, and lidar.

## 1. Introduction

Continued advances in optical imaging and the efficient use of clean energy place increasingly stringent demands on the performance of optical components [1,2]. Fresnel reflection at optical surfaces is a major factor limiting optical-system throughput. In complex systems containing multiple lenses or windows, cumulative optical-energy losses can exceed 30%, substantially degrading the imaging signal-to-noise ratio and detection sensitivity [3]. The visible-to-near-infrared region is the principal operating range of silicon-based detectors, optical-fiber communications, lidar, and related devices, and reflection losses occur throughout this range. By suppressing surface reflection through destructive interference, antireflection coatings provide an essential means of improving optical throughput [4,5].

Previous studies have optimized single- and multilayer antireflection designs for solar-cell applications [6,7] and prepared visible or near-infrared coatings on glass and CaF_2_ substrates by electron-beam deposition [8,9]. Hybrid MgF_2_/SiO_2_ structures and solution-derived oxide coatings have also been investigated to broaden the operating band or introduce additional surface functions [10,11,12,13], while multiband infrared and crystalline-silicon coating designs demonstrate the flexibility of multilayer interference control [14,15]. These reports establish the effectiveness of material and structural optimization. However, most previous studies have focused on one or two of these aspects individually, and an integrated workflow that combines deposition-condition-dependent refractive-index calibration, layer-specific thickness-sensitivity and tolerance analysis, and multi-batch performance validation has rarely been reported for broadband antireflection coatings. In this work, we demonstrate such a design-to-fabrication workflow for a double-sided seven-layer broadband antireflection coating. The obtained coating shows promising performance for use in imaging systems, optical windows, and lidar applications.

## 2. Design Principles and Characterization Methods

### 2.1. Interference Principle of Antireflection Coatings

Antireflection coatings suppress reflection losses from optical surfaces through thin-film interference. When light is incident on a film, reflections are generated at the upper and lower interfaces, and the two reflected waves interfere because of their optical-path difference. For a single dielectric layer, destructive interference occurs when its optical thickness satisfies

(1) $$nd=\lambda_{0}/4$$

where *n* is the refractive index of the film; *d* is its physical thickness; and *λ*_0_ is the vacuum wavelength. Under this condition, the optical-path difference between the reflections at the two interfaces is *λ*_0_/2, corresponding to a phase difference of π. If the refractive index of the layer also satisfies

(2) $$n=\sqrt{\left(n_{0}n_{s}\right)}$$

where *n*_0_ and *n*_s_ are the refractive indices of the incident medium (air) and the substrate, respectively, the reflectance of a single-layer coating can theoretically approach zero at the center wavelength *λ*_0_.

In practice, materials that exactly satisfy this index-matching condition are difficult to obtain. For a K9 glass substrate, the required index is approximately 1.23, whereas MgF_2_, a commonly used low-index material, has an index of approximately 1.38. A single MgF_2_ layer therefore cannot eliminate reflection completely. Moreover, a single-layer coating provides strong antireflection performance only near its center wavelength; reflectance rises rapidly away from that wavelength, limiting the usable bandwidth.

Stable antireflection performance over a broad spectral range requires a multilayer architecture. Alternating high- and low-index layers enable destructive interference over an extended bandwidth. According to the equivalent-interface principle, the multilayer stack can be treated as an effective interface whose reflectance is kept low across the target band by appropriately adjusting the thickness of each layer.

### 2.2. Material Selection

Material selection is critical to both the optical performance and structural stability of broadband coatings for the visible and near-infrared regions. Refractive-index matching, interlayer stress compatibility, spectral transparency, and chemical stability must all be considered. Materials must not only possess favorable optical properties but also remain physically and chemically compatible within the multilayer structure to ensure reliable operation under different application conditions. Based on these criteria, Al_2_O_3_ [16] was selected as the transition layer, TiO_2_ [17] as the high-index material, and SiO_2_ [18] and MgF_2_ [19] as the low-index materials. K9 glass was used as the substrate.

### 2.3. Characterization Methods

Transmittance measurements were performed using an ultraviolet–visible–near-infrared (UV-Vis-NIR) spectrophotometer (UV-3600i Plus, Shimadzu, Kyoto, Japan) with the incident beam aligned normal to the sample surface (0° incidence). Before each measurement, an air-reference baseline scan was performed. The initially characterized sample was measured over 400–1100 nm with a scanning step of 1 nm. To assess sample-to-sample consistency, four coating samples from one additional deposition batch, prepared using the same multilayer design and process parameters, were measured. Statistical analysis was conducted over the 400–1100 nm range. At each wavelength, the mean and sample standard deviation were calculated across the four samples from the same batch. The band-average transmittance of each sample was also summarized as mean ± sample standard deviation.

Surface morphology and cross-sectional structure were examined by scanning electron microscopy (SEM; JEOL Ltd., Tokyo, Japan). The specimens were sputter-coated with gold to improve electrical conductivity. An accelerating voltage of 5.0 kV and magnifications of 40,000× and 100,000× were used to obtain nanoscale structural images. For EDS analysis, a separate cross-sectional specimen was coated with platinum to avoid overlap with the gold signal, and a regional survey spectrum was collected from the marked area (Spectrum 15) to confirm the elemental composition of the coating cross-section. The platinum coating was excluded from the reported semi-quantitative results.

The total film thickness was measured nondestructively with a step profiler (JS10B, Anhui Zeyou Technology Co., Ltd., Tongling, China) having a vertical resolution of ±0.5 nm. Before deposition, a small region near the substrate edge was covered with a mask to create a distinct step. After deposition, profiles were acquired perpendicular to the step at no fewer than five positions, and the mean value was reported to minimize errors caused by local nonuniformity.

## 3. Coating Design and Fabrication

### 3.1. Deposition Parameters for Single-Layer Films

Refractive index is one of the most important parameters in optical thin films, and its accurate determination is essential for evaluating and optimizing coating performance. Because the optical properties of a material vary with deposition conditions, the optimal processing window for each single-layer film was determined before fabrication of the multilayer stack. All single-layer samples were deposited by ion-assisted electron-beam evaporation. The chamber base pressure was 5 × 10^−4^ Pa, and the substrate temperature was maintained at 300 °C during deposition.

For TiO_2_ and SiO_2_, a controlled-variable approach was used to investigate three key parameters: ion-source current, oxygen flow rate, and deposition rate. Refractive indices were calculated from spectrophotometric transmittance spectra using an envelope method based on interference-fringe extrema [20]. The deposition parameters for Al_2_O_3_ were adopted from our previous work, while those for MgF_2_ followed the standard equipment recipe. The optimal parameters for all four materials are summarized in Table 1.

**Figure 1:**
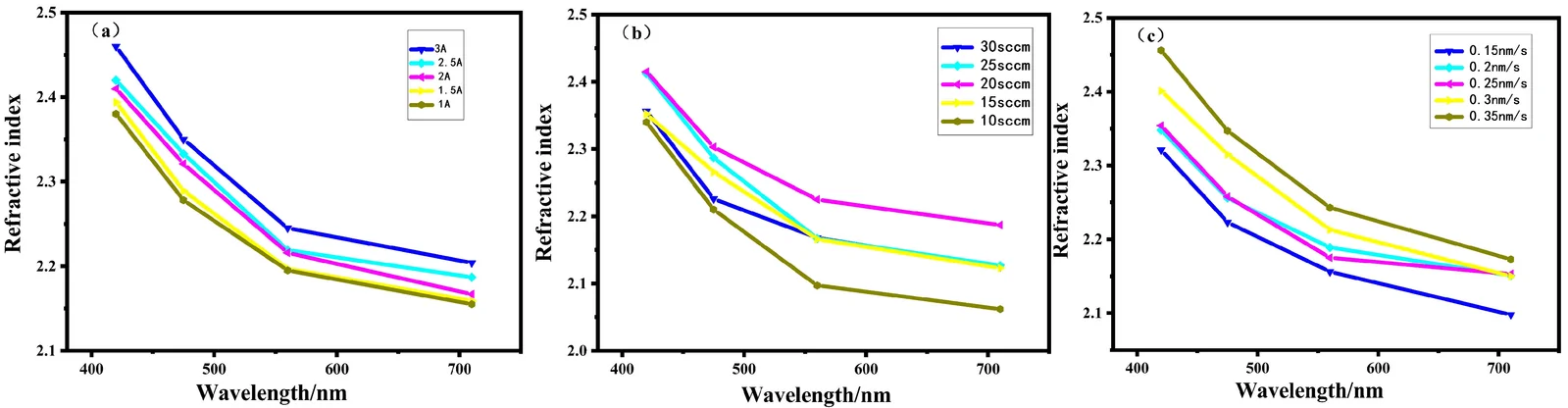
Refractive-index variation in TiO_2_ single-layer films under different deposition parameters: (**a**) ion-source current; (**b**) oxygen flow rate; and (**c**) deposition rate.

Figure 1 shows the refractive index of TiO_2_ single-layer films deposited under different conditions. The ion-source current was varied from 1 to 3 A in 0.5 A increments, the oxygen flow rate from 10 to 30 standard cubic centimeters per minute (sccm) in 5 sccm increments, and the deposition rate from 0.15 to 0.35 nm s^−1^ in 0.05 nm s^−1^ increments.

**Figure 2:**
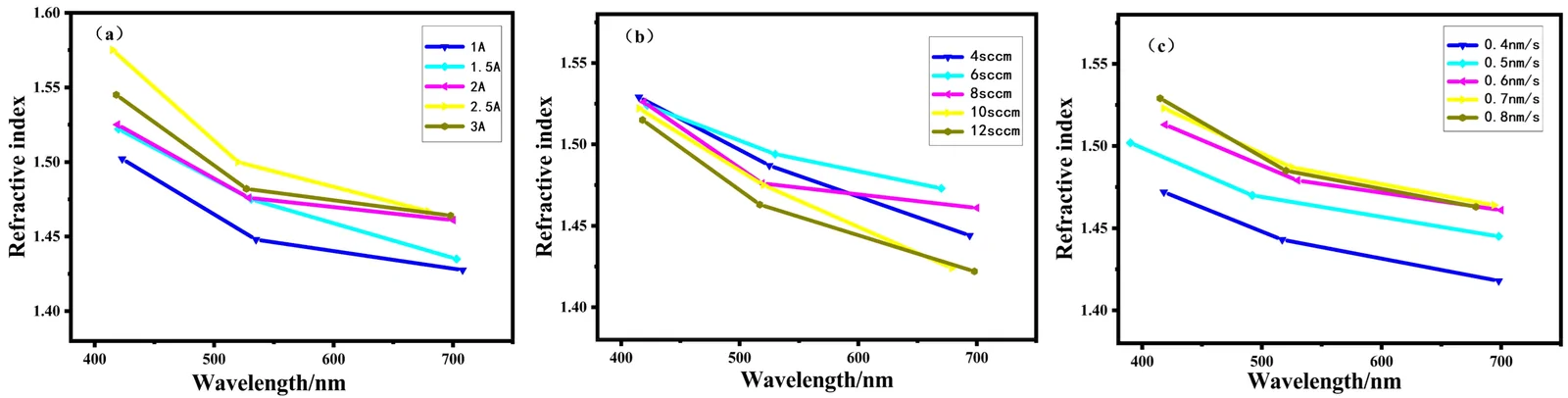
Refractive-index variation in SiO_2_ single-layer films under different deposition parameters: (**a**) ion-source current; (**b**) oxygen flow rate; and (**c**) deposition rate.

Figure 2 shows the corresponding refractive-index variation in SiO_2_ single-layer films. The ion-source current was varied from 1 to 3 A in 0.5 A increments, the oxygen flow rate from 4 to 12 sccm in 2 sccm increments, and the deposition rate from 0.4 to 0.8 nm s^−1^ in 0.1 nm s^−1^ increments.

### 3.2. Design and Optimization of the Antireflection Coating

The initial broadband antireflection design was guided by Willey’s empirical formula. The periodic starting structure was Sub|M 0.3H 0.3L 2H 0.3L 0.3H F|Air, where H, L, M, and F represent quarter-wave optical thicknesses of TiO_2_, SiO_2_, Al_2_O_3_, and MgF_2_, respectively, at a reference wavelength of 550 nm.

The initial design was subsequently refined in TFCalc using needle optimization and gradual evolution. Individual layer thicknesses were optimized to maximize average transmittance from 400 to 1100 nm while controlling spectral ripple. The resulting seven-layer structure and optimized thicknesses are listed in Table 2.

**Figure 3:**
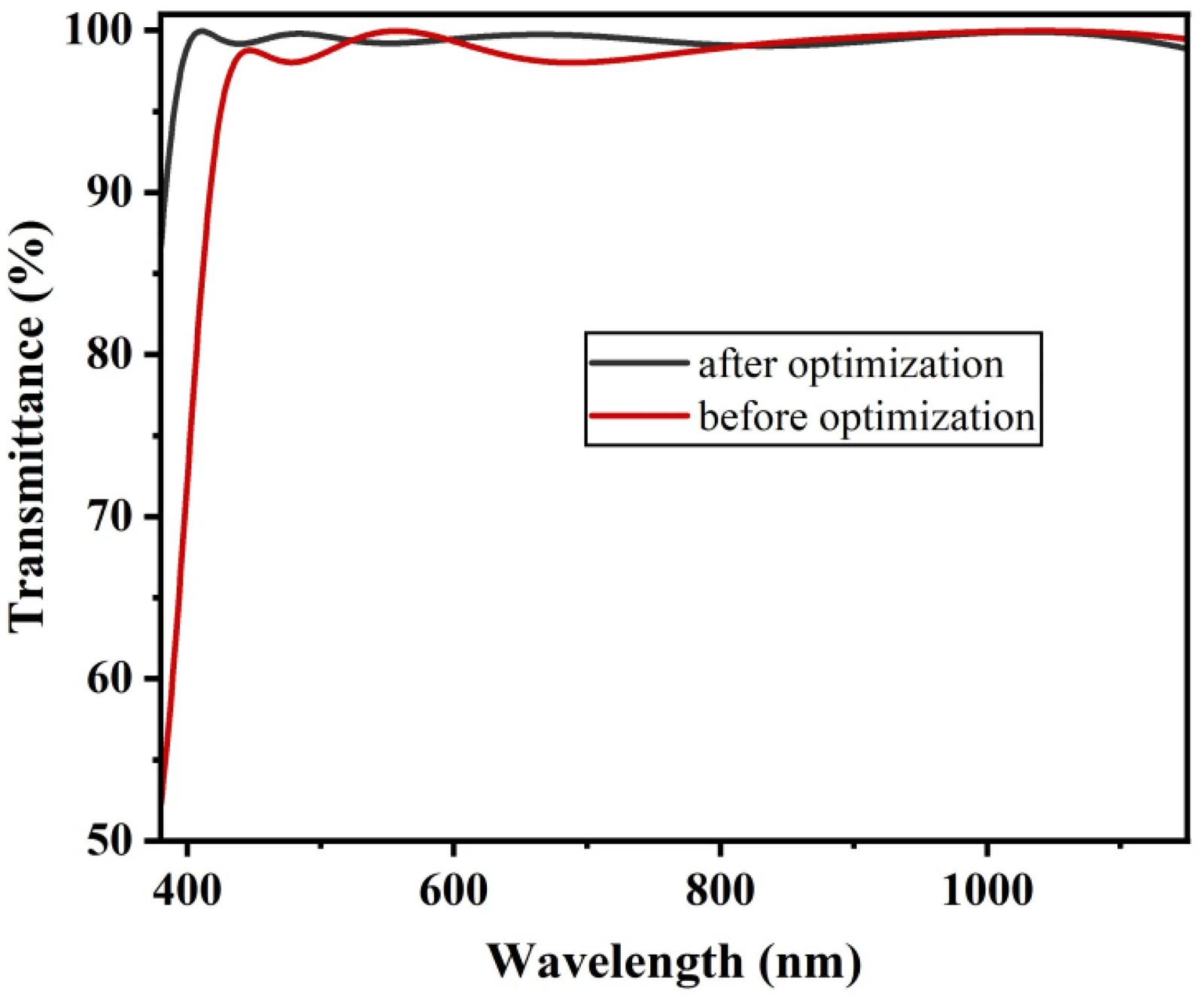
Calculated transmittance spectra of the broadband antireflection coating before and after optimization over 400–1100 nm.

Before optimization, the transmittance spectrum fluctuated strongly: the maximum and minimum transmittances were 99.972% and 72.330%, respectively, and the average transmittance was 98.595%. Optimization markedly improved spectral uniformity. The minimum transmittance increased to 98.967%, the average transmittance to 99.495%, and the maximum transmittance to 99.949%. As shown in Figure 3, the improvement was particularly pronounced in the visible region from 400 to 780 nm.

### 3.3. Sensitivity Analysis of Thickness Errors

**Figure 4:**
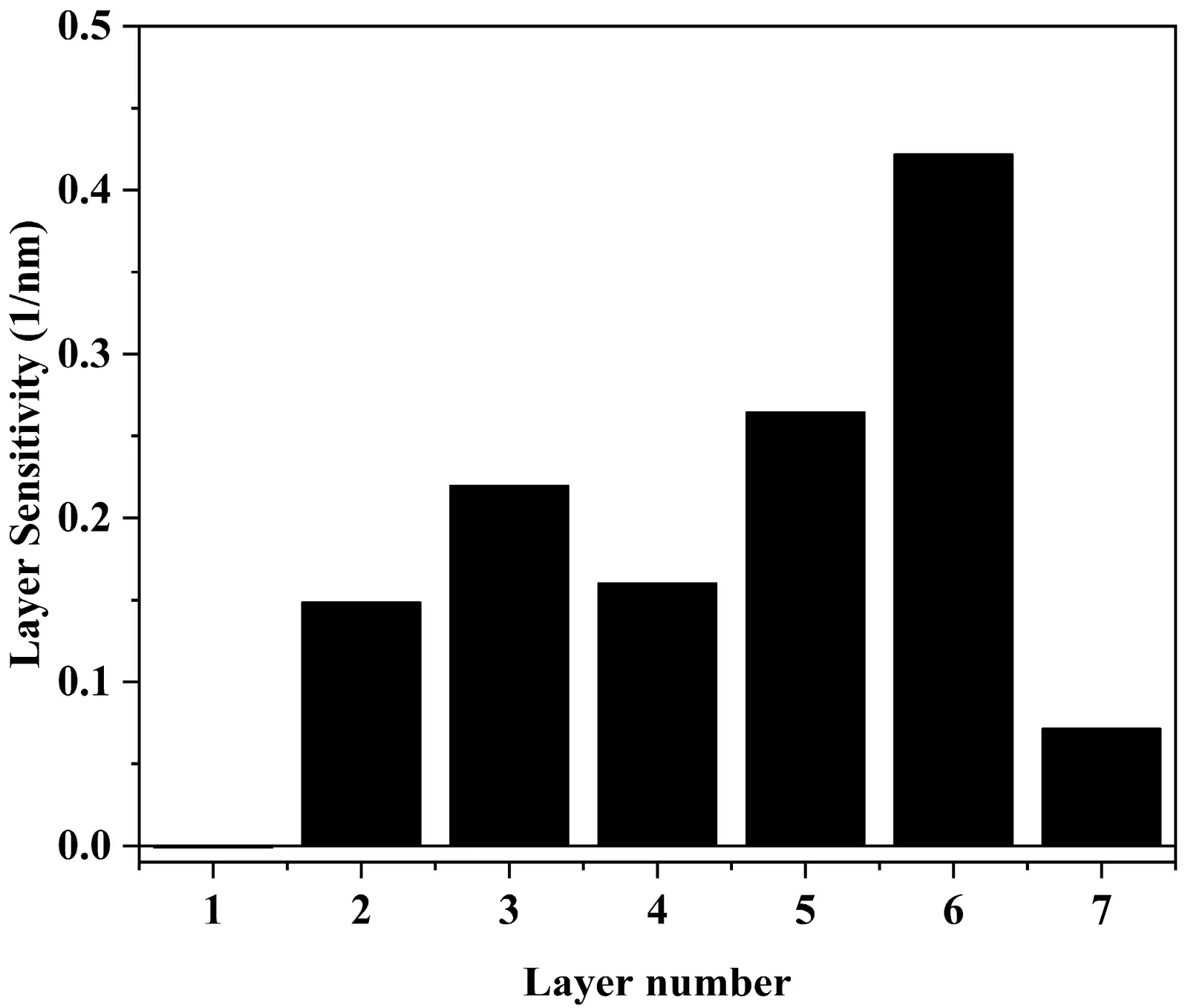
Second-order thickness sensitivity distribution of each layer for the seven-layer antireflection coating structure.

In practical deposition operations, the actual thickness of each layer hardly matches the theoretical design value perfectly [21]. In addition, unavoidable deviations are induced by equipment operating disturbances during coating fabrication. The second-order thickness sensitivity of each layer was calculated using TFCalc, as presented in Figure 4, where the amplitude of each bar quantifies the impact of thickness fluctuations on optical properties. Within this seven-layer antireflection coating stack, the 6th layer possesses the maximum sensitivity and serves as the critical sensitive layer. Real-time, high-precision thickness monitoring should be implemented for this layer during electron beam evaporation to mitigate spectral degradation originating from thickness drift.

**Figure 5:**
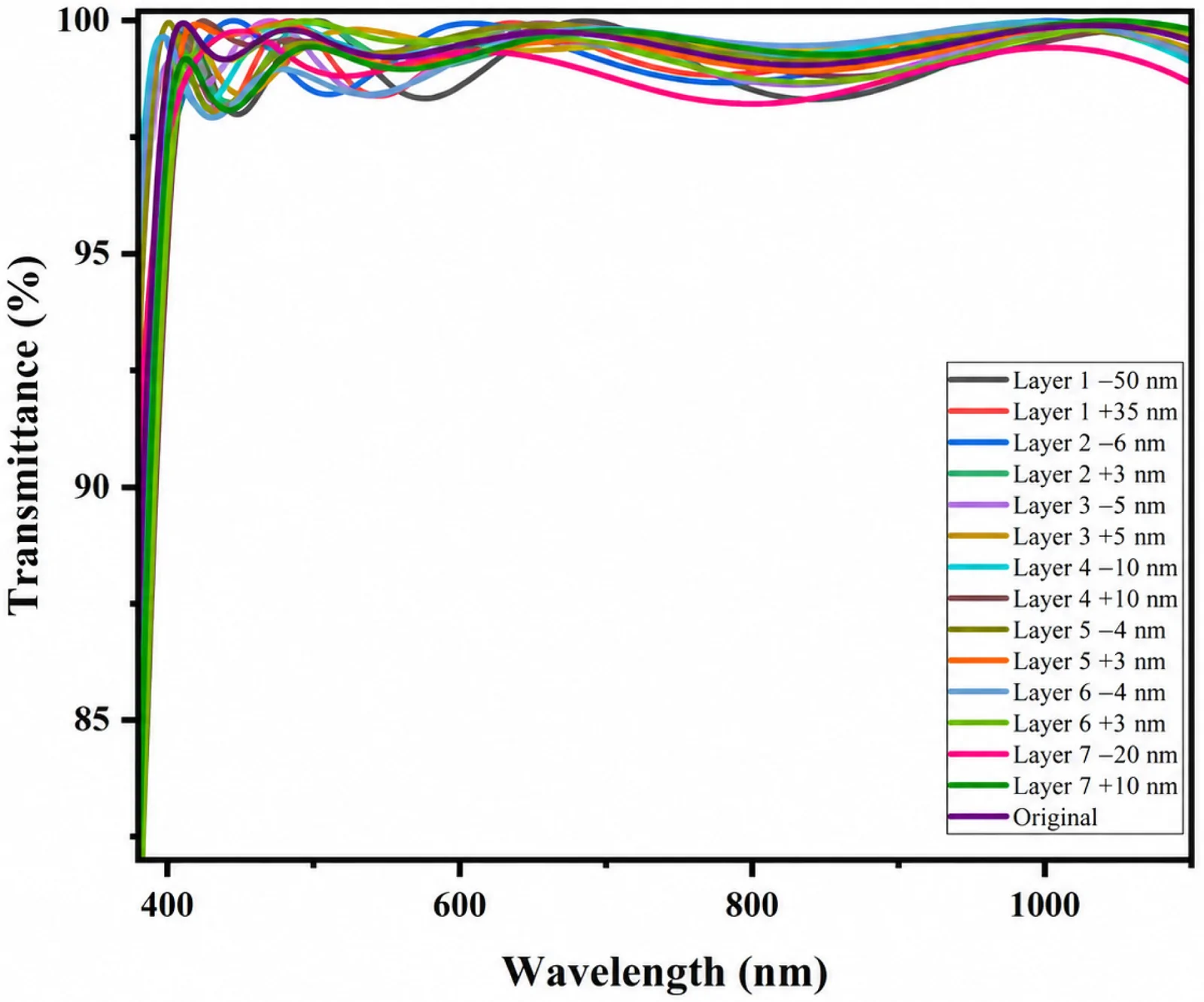
Transmittance spectra corresponding to thickness variations in each individual layer in the broadband antireflection coating.

Positive and negative thickness perturbations were applied to individual layers to confirm the allowable process tolerance range, within which minor thickness errors would not induce obvious deterioration of antireflection characteristics. Two quantitative indicators were formulated to define the valid tolerance window: the average transmittance over the entire 400–1100 nm band should be higher than 99%, and the minimum transmittance in the operating wavelength region ought to stay above 98% to suppress intense spectral oscillations. Figure 5 records the transmittance spectral responses when separate thickness deviations are introduced in each film layer. The simulation results reveal that the valley transmittance value is highly sensitive to thickness drift, and spectral distortion is most pronounced within the short-wavelength region. Spectral blue shift generally occurs with increased physical thickness for most layers, while thinner deposition contributes to a red shift in the transmission curve. According to the spectral variation trends under different perturbation magnitudes, the feasible thickness tolerance for each layer is sorted out and listed in Table 3. The sixth layer, identified as the most sensitive layer in the previous sensitivity calculation, possesses the narrowest tolerance interval of −2 nm to +3 nm and requires rigorous thickness control during deposition. By contrast, Layer 3 and Layer 4 exhibit favorable fabrication robustness with allowable deviations of ±5 nm and ±10 nm, respectively. Excessive negative thickness offsets of Layer 1 and Layer 7 will greatly worsen transmission performance, so negative thickness drift should be restricted during actual film growth.

### 3.4. Incident-Angle Dependence

**Figure 6:**
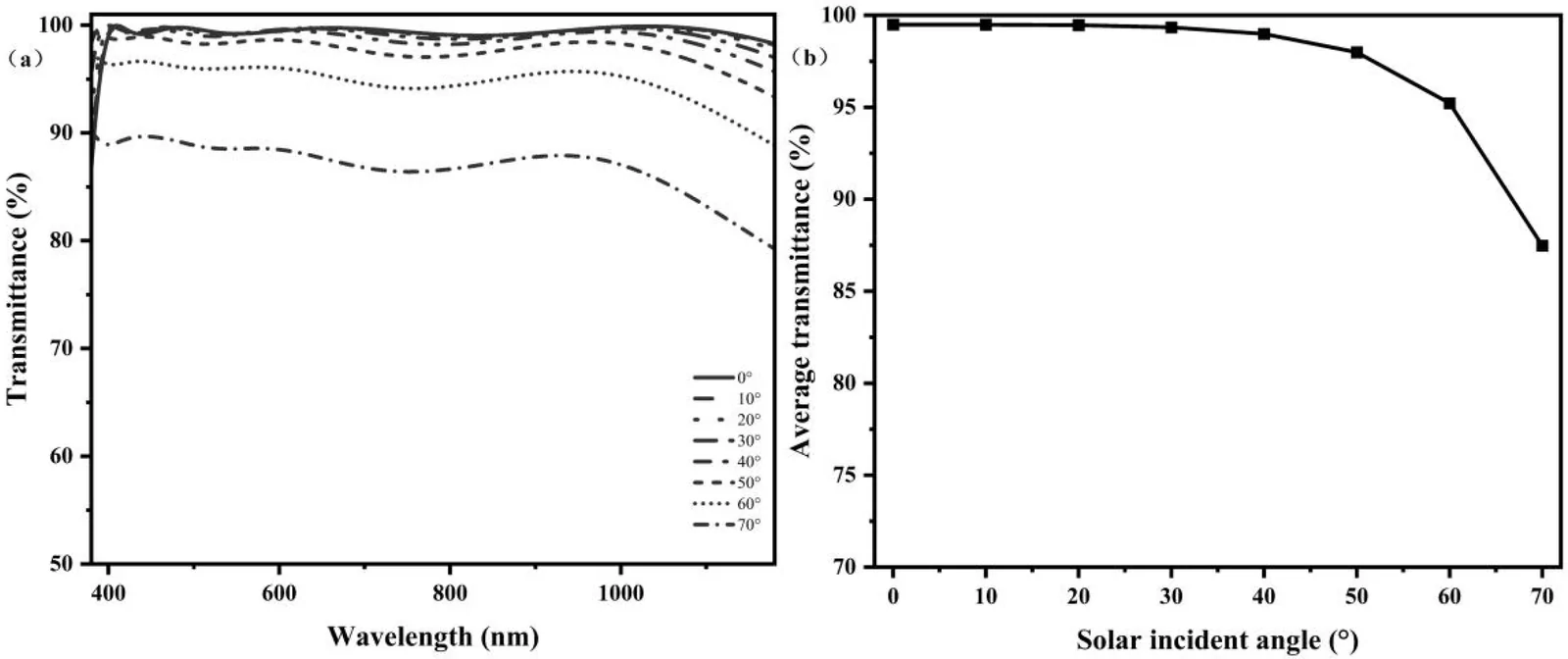
Spectral transmittance of the coating at different incidence angles: (**a**) transmittance spectra at each angle; (**b**) average transmittance as a function of incidence angle.

In practical optical systems, light does not always strike the film surface at normal incidence. For imaging lenses, off-axis rays inherently propagate at large angles, particularly in wide-field or wide-angle systems where the incident angle at the edge of the field of view can reach 40° or more. Even for planar optical windows, oblique incidence inevitably occurs when the mounting angle or the direction of incident light deviates from the surface normal. Consequently, investigating the optical response of antireflection coatings under varying incident angles is of practical significance for evaluating their performance in real-world applications [22]. Figure 6 compares the spectral transmittance at different angles: Figure 6a shows the spectra, and Figure 6b shows the corresponding average transmittance.

At near-normal incidence from 0° to 30°, the 400–1100 nm spectra nearly overlap, and the average transmittance remains above 99%, indicating excellent angular stability. At 40–50°, transmittance decreases slightly, but the average remains above 98%. Pronounced attenuation occurs beyond 60°, and the average transmittance decreases to approximately 87.488% at 70°. This behavior arises from the change in effective refractive index, polarization effects, and the shift in optical thickness under oblique incidence, which disrupt the conditions optimized for normal incidence. Nevertheless, high transmittance is retained over the broad angular range from 0° to 60°, supporting applications such as optical windows subject to dynamically varying incidence.

### 3.5. Fabrication of the Antireflection Coating

The optimized structure was deposited on both sides of K9 glass substrates. Before deposition, the substrates were ultrasonically cleaned for 15 min each in acetone, absolute ethanol, and deionized water, and then dried in an oven at 120 °C for 2 h. The optimal conditions established in the single-layer experiments were used. Layer thickness was monitored in real time with a quartz-crystal oscillator. Deposition was performed in a ZZS720 electron-beam vacuum coater (Chengdu Xingnan Technology Co., Ltd., Chengdu, China) equipped with an auxiliary ion source. Ion cleaning was applied before deposition, and continuous ion bombardment during growth improved the uniformity and density of the layers. After all layers had been deposited, the substrate was allowed to cool naturally to room temperature under vacuum to prevent thermally induced cracking. The sample was then removed, inverted, and coated on the second side under the same conditions.

## 4. Results and Discussion

### 4.1. Optical Performance

**Figure 7:**
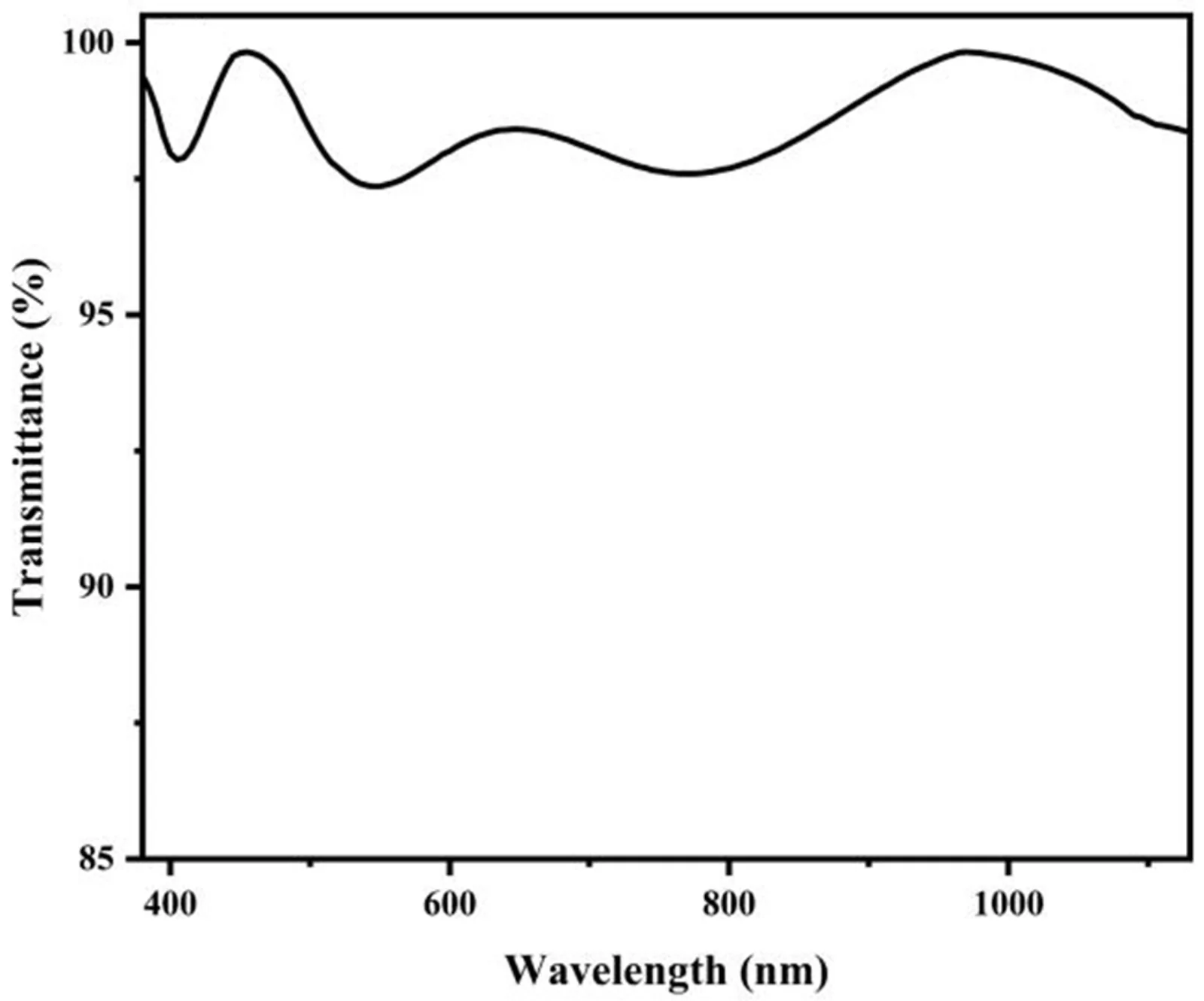
Measured transmittance of the initially characterized double-sided broadband antireflection coating over 400–1100 nm.

The measured transmittance of the initially characterized double-sided sample is shown in Figure 7. Over 400–1100 nm, the average transmittance was 98.72%, with a maximum of 99.83% at 970 nm and a minimum of 97.85% at 405 nm. This result demonstrates high transmission through the double-sided coated substrate over a broad spectral range. To assess sample-to-sample uniformity, four samples from a separate deposition batch were measured under the same conditions. Their transmittance spectra closely overlapped across the entire 400–1100 nm range, with a mean band-average transmittance of approximately 98.657%. This batch mean agreed well with the value of the initial sample (98.72%), supporting the consistency of the deposition process. The corresponding spectra and statistics are provided in Supplementary Figure S1 and Supplementary Table S1.

### 4.2. Microstructural Characterization

**Figure 8:**
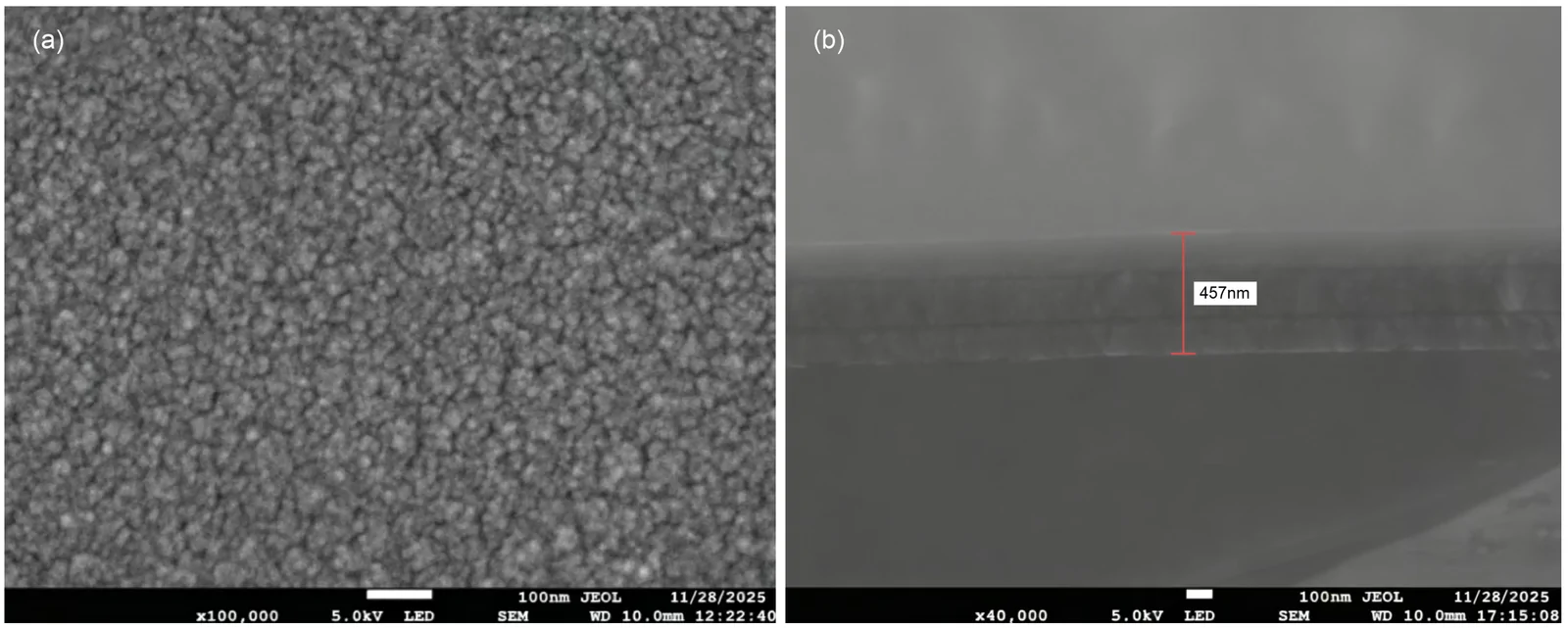
Scanning electron microscopy images of the broadband antireflection coating: (**a**) surface morphology; (**b**) cross-sectional morphology.

The surface and cross-sectional morphologies were examined by SEM (Figure 8). The surface image in Figure 8a shows a relatively smooth and dense coating; no obvious pinholes, cracks, or anomalously large particle agglomerates are visible within the examined field. The cross-sectional image in Figure 8b shows continuous multilayer contrast and distinguishable interfaces at the spatial resolution of SEM, with no obvious interfacial cracking in the observed region. A regional EDS survey from the marked Spectrum 15 area detected O, F, Mg, Al, Si, and Ti (Figure S2 and Table S2), consistent with the constituent elements of the coating materials and K9 substrate. Because the acquisition area included multiple adjacent layers, the EDS composition represents the combined analyzed region.

**Figure 9:**
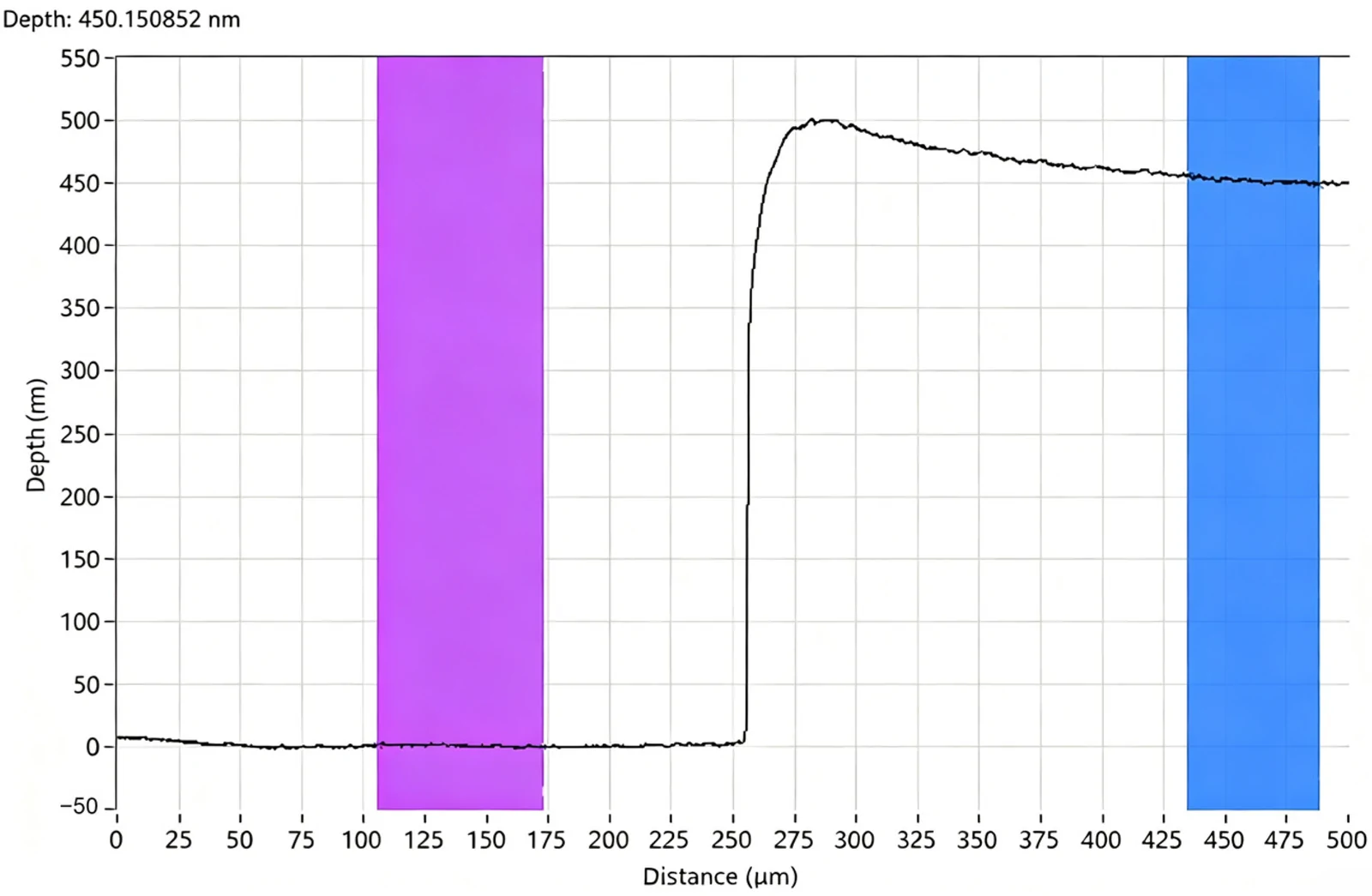
Step profiler scanning curve measured at the lower-left position of the sample. The purple and blue shaded regions indicate the uncoated-substrate reference region and the coated-surface measurement region used to determine the step height, respectively.

A representative step-profile curve measured at the lower-left position is presented in Figure 9. A well-defined step between the film and substrate is visible. Five-position measurements are summarized in Table 4. The measured total thickness of one seven-layer stack ranged from 448.03 to 454.82 nm, with a mean of 451.41 ± 2.52 nm (mean ± standard deviation (SD), *n* = 5) and a relative standard deviation of 0.56%. The mean value was 0.42 nm lower than the designed total thickness of 451.83 nm, while the individual deviations ranged from −3.80 to +2.99 nm. These results indicate reasonable agreement between the measured mean total thickness and the design, together with good total-thickness uniformity across the five measured positions.

## 5. Conclusions

In this study, a seven-layer broadband antireflection coating consisting of TiO_2_, SiO_2_, Al_2_O_3_, and MgF_2_ was designed and fabricated for the spectral region of 400–1100 nm. The primary sample achieves an average transmittance of 98.72% over the target band. Four specimens from another independent deposition batch show highly consistent transmittance spectra with a band-average transmittance of 98.66%, demonstrating satisfactory intra-batch uniformity and acceptable batch-to-batch consistency. Five-point profilometry returns a total coating thickness of 451.41 ± 2.52 nm (*n* = 5, relative standard deviation (RSD) = 0.56%), matching the designed value of 451.83 nm. Cross-sectional SEM displays continuous multilayer morphology, and regional EDS confirms the expected constituent elements. These findings illustrate the application potential of such broadband antireflection coatings for visible-to-near-infrared optical windows, imaging systems, and lidar components.

## Figures and Tables

**Table 1:** Optimal deposition parameters for the single-layer films.

Material	Ion Current (A)	O_2_ Flow (sccm)	Deposition Rate (nm·s^−1^)	Temp. (°C)	Base Pressure (Pa)
TiO_2_	3	20	0.3	300	5 × 10^−4^
SiO_2_	2	8	0.6	300	5 × 10^−4^
Al_2_O_3_	2	20	0.7	300	5 × 10^−4^
MgF_2_	2	0	1.0	300	5 × 10^−4^

**Table 2:** Layer thicknesses of the seven-layer broadband antireflection coating before and after optimization.

Layer No.	Material	Pre-Optimized Thickness (nm)	Post-Optimized Thickness (nm)
1 (substrate side)	Al_2_O_3_	105.11	100.16
2	TiO_2_	21.88	21.91
3	SiO_2_	34.70	21.89
4	TiO_2_	145.90	141.68
5	SiO_2_	34.70	27.74
6	TiO_2_	21.88	21.82
7 (air side)	MgF_2_	123.43	116.63

**Table 3:** Allowable thickness tolerance for each layer of the seven-layer antireflection coating.

Layer Number	Allowable Thickness Tolerance (nm)
Layer 1	−10~+10
Layer 2	−3~+3
Layer 3	−5~+5
Layer 4	−10~+10
Layer 5	−4~+3
Layer 6	−2~+3
Layer 7	−5~+10

**Table 4:** Thickness values of the thin film measured by step profiler at five sampling positions.

Sampling Position	Measured Thickness (nm)	Deviation from Designed Value (nm)
Center	452.21	+0.38
Top-Left	454.82	+2.99
Top-Right	448.03	−3.80
Bottom-Left	450.15	−1.68
Bottom-Right	451.84	+0.01
Average value of five points	451.41	−0.42
Designed thickness	451.83	—
Standard deviation (*n* = 5)	2.52	—
Relative standard deviation	0.56%	—

## Data Availability

The original contributions presented in this study are included in the article/Supplementary Materials. Further inquiries can be directed to the corresponding author.

## Supplementary Materials

The following supporting information can be downloaded at: https://www.mdpi.com/article/10.3390/mi17091077/s1, Figure S1: Transmittance spectra of four broadband antireflection-coated samples prepared in one additional deposition batch. The figure shows the individual spectra (Samples 1–4), the wavelength-dependent mean, and the shaded mean ± sample SD (*n* = 4); the inset uses an enlarged ordinate scale; Table S1: Band-average transmittance statistics for four samples prepared in one additional deposition batch over 400–1100 nm; Figure S2: Cross-sectional area selected for the regional EDS survey analysis and the corresponding Spectrum 15; Table S2: Semi-quantitative elemental composition obtained from Spectrum 15, excluding the platinum conductive coating.

## Abbreviations

The following abbreviations are used in this manuscript:

AR coating	Antireflection coating
SEM	Scanning electron microscopy
sccm	Standard cubic centimeter per minute
UV-Vis-NIR	Ultraviolet–visible–near-infrared
EDS	Energy-dispersive X-ray spectroscopy
SD	Standard deviation
RSD	Relative standard deviation
