# Supplementary material for: Preparation and Performance Investigation of Broadband Antireflection Coatings in the Visible and Near-Infrared Spectral Regions
Source: Micromachines (Basel). 2026 Sep 11;17(9):1077. doi: 10.3390/mi17091077 (PMC13609015; doi:10.3390/mi17091077)
Supplement: Supplementary file 1 [file micromachines-17-01077-s001.zip › micromachines-4514191-supplementary.pdf]

Supplementary Information

Preparation and Performance Investigation of Broadband Antireflection Coatings in the Visible and Near-Infrared Spectral Regions

Within-batch sample-to-sample consistency analysis. Four coating samples were prepared in one additional deposition batch using the same seven-layer design and deposition parameters; this batch was separate from that of the initially characterized sample reported in the main text. Transmittance spectra were recorded separately over 380–1120 nm at 5 nm intervals, and statistics were calculated over 400–1100 nm. At each wavelength, the arithmetic mean and sample standard deviation ($n = 4$; denominator $n - 1$) were calculated. Band-average transmittance was also calculated separately for each sample over the common 400–1100 nm analysis range.

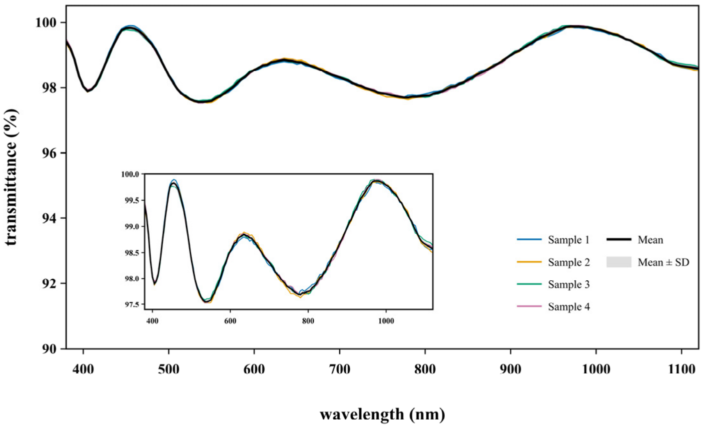

Figure S1. Transmittance spectra of four broadband antireflection-coated samples prepared in one additional deposition batch. The figure shows the individual spectra (Samples 1–4), the wavelength-dependent mean, and the shaded mean ± sample SD ($n = 4$); the inset uses an enlarged ordinate scale.

Table S1. Band-average transmittance statistics for four samples prepared in one additional deposition batch over 400–1100 nm.

Sample	Average transmittance (%)	Minimum transmittance (%)	Maximum transmittance (%)
Sample 1	98.659	97.54	99.90
Sample 2	98.653	97.53	99.83
Sample 3	98.660	97.59	99.89
Sample 4	98.656	97.52	99.87

The four band-average transmittances yielded 98.657 ± 0.002% (mean ± sample SD, $n = 4$). This value describes the dispersion of the band averages among samples from the same deposition batch; it is not a measurement uncertainty or an estimate of run-to-run reproducibility. For the spectral curves, the mean wavelength-wise sample SD over 400–1100 nm was 0.0367 percentage points, and the maximum wavelength-wise sample SD was 0.0660 percentage points at 950 nm. These statistics indicate low sample-to-sample variation within the examined batch.

Regional EDS analysis. A separate cross-sectional specimen was coated with platinum for the supplementary EDS measurement. A survey spectrum was collected from the area marked Spectrum 15, which covered multiple adjacent layers of the coating cross-section. Platinum from the conductive coating was excluded from the reported semi-quantitative composition.

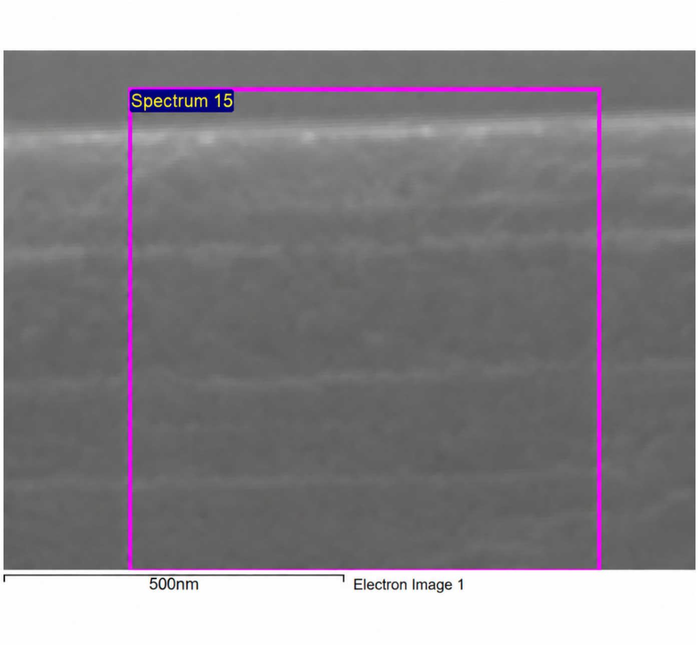

(a) Cross-sectional area selected for Spectrum 15

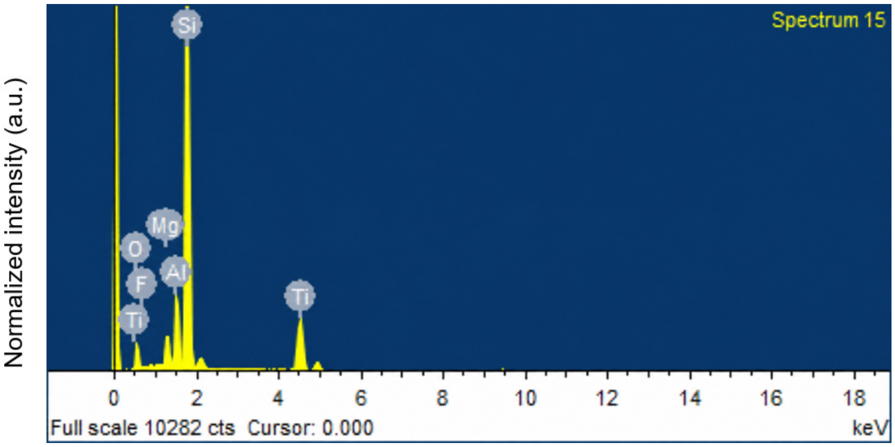

(b) EDS survey spectrum from the selected area

Figure S2. Cross-sectional area selected for the regional EDS survey analysis and the corresponding Spectrum 15.

Table S2. Semi-quantitative elemental composition obtained from Spectrum 15, excluding the platinum conductive coating.

Region	Element	Weight (%)	Atomic (%)
Spectrum 15	O K	25.76	38.69
	F K	2.59	3.28
	Mg K	3.63	3.58
	Al K	7.77	6.92
	Si K	48.91	41.84
	Ti K	11.34	5.69

O, F, Mg, Al, Si, and Ti were detected in the selected cross-sectional area, consistent with the constituent elements of the coating materials and K9 substrate. Because the acquisition area covered multiple adjacent layers, the reported composition represents the combined analyzed region rather than individual layers.
